# Hospital Meetings

**Published:** 1906-02-24

**Authors:** 


### HOSPITAL MEETINGS.

### WEST-END HOSPITAL.

The annual meeting of the Governors and subscribers of the West End Hospital was held on February 16.

The chair was taken by Sir Lennox Napier, who, in moving the adoption of the report, said that the committee had to deplore the loss of their late Consulting Surgeon, Mr. Christopher Heath, who had always been a kind friend to the hospital, and whose death came as a sudden blow to them. They had succeeded in obtaining the services of Mr. John Langton to fill the vacancy. Dr. A. de Watteville had resigned his post during the last year, and Mr. Harry Campbell had been appointed to be in-patent physician in his place.

For some years past they had been trying to make provision for the extension of the hospital, in order to give accommodation for adult patients, and the subject had been very seriously considered. There were large numbers of out-patients who could only be successfully treated by being taken into the hospital. They had now secured a house, and hoped in the course of a short time to have it open, where they could take in 25 adult in-patients. The site they had obtained would perhaps enable them to concentrate the hospital under one roof, an arrangement much to be desired, as at the present time, including their new house, they were established under three roofs.

With regard to finances, they were very glad to see that last year there had been an increase of income over the previous year. In 1904 their income was £2,557, while in 1905 it was £2,813; a considerable advance when the prevalence of bad times was borne in mind.

The adoption of the report having been seconded by Dr. T. Outterson Wood and passed, the customary votes of thanks were passed.

